# Rates of Vaccine Evolution Show Strong Effects of Latency: Implications for Varicella Zoster Virus Epidemiology

**DOI:** 10.1093/molbev/msu406

**Published:** 2015-01-06

**Authors:** Lucy A. Weinert, Daniel P. Depledge, Samit Kundu, Anne A. Gershon, Richard A. Nichols, Francois Balloux, John J. Welch, Judith Breuer

**Affiliations:** ^1^Department of Veterinary Medicine, University of Cambridge, Cambridge, United Kingdom; ^2^Department of Genetics, Evolution and Environment, UCL, London, United Kingdom; ^3^Division of Infection and Immunity, MRC Centre for Medical Molecular Virology, UCL, London, United Kingdom; ^4^Division of Infectious Disease, Columbia University Medical Centre, New York, USA; ^5^School of Biological and Chemical Sciences, Queen Mary University of London, London, United Kingdom; ^6^Department of Genetics, University of Cambridge, Cambridge, United Kingdom

**Keywords:** within-patient evolution, whole-genome sequencing, molecular dating

## Abstract

Varicella-zoster virus (VZV) causes chickenpox and shingles, and is found in human populations worldwide. The lack of temporal signal in the diversity of VZV makes substitution rate estimates unreliable, which is a barrier to understanding the context of its global spread. Here, we estimate rates of evolution by studying live attenuated vaccines, which evolved in 22 vaccinated patients for known periods of time, sometimes, but not always undergoing latency. We show that the attenuated virus evolves rapidly (∼10^−6^ substitutions/site/day), but that rates decrease dramatically when the virus undergoes latency. These data are best explained by a model in which viral populations evolve for around 13 days before becoming latent, but then undergo no replication during latency. This implies that rates of viral evolution will depend strongly on transmission patterns. Nevertheless, we show that implausibly long latency periods are required to date the most recent common ancestor of extant VZV to an “out-of-Africa” migration with humans, as has been previously suggested.

## Introduction

Varicella-zoster virus (VZV; also known as human herpesvirus 3) is a nuclear-replicating double-stranded DNA (dsDNA) virus, with a genome of around 125 kb and over 70 open reading frames. The virus is the causative agent of varicella (chicken pox), and herpes zoster (shingles), two conditions that can lead to serious complications (World Health Organization 1998; Gilden et al. 2011).

The global genetic diversity of VZV is now well characterized, with five major clades segregating geographically (Muir et al. 2002; Schmidt-Chanasit and Sauerbrei 2011; Grose 2012; Zell et al. 2012; Chow et al. 2013). But although its phylogeny is well resolved, there is less certainty about the age of VZV’s most recent common ancestor, and this is a major impediment to understanding its origins and the ecological context of its global spread.

For example, Zell et al. (2012) analyzed VZV along with other alphaherpesvirinae (see also McGeoch and Cook 1994; Muir et al. 2002; Grose 2012), and showed that the viral phylogeny agreed exactly with the phylogeny of their mammalian hosts (Meredith et al. 2011), suggestive of host-virus cospeciation. Calibration of the viral phylogeny with the mammalian fossil record placed the most recent common ancestor of VZV at approximately 110,000 years ago. This date is consistent with the prominent theory that VZV spread around the globe by accompanying humans on their out-of-Africa migrations (Grose 1999, 2012; Petraglia et al. 2010)—a pattern also observed in other pathogens (Moodley et al. 2012; Paraskevis et al. 2013). In contrast, Firth et al. (2010) analyzed viruses sampled from around the world over a 37-year period, and, using a method of dated tips (Rambaut 2000), estimated that the most recent common ancestor of VZV lived just 309 years ago (95% highest posterior density [HPD] 51–741).

Such wildly different rate estimates have been remarkably common in the study of viruses, suggesting that something is severely wrong with our assumptions about their evolution (Holmes 2003; Sharp and Simmonds 2011; Lythgoe and Fraser 2012). In fact, as the authors recognize, there are good reasons to doubt both previous estimates for the age of VZV. Specifically, Firth et al.’s (2010) method of dated tips relies on data sets showing sufficient temporal signal, but despite VZV samples obtained over 37 years, randomization of the sampling dates provided similar estimates, suggesting that true temporal signal was absent. In contrast, estimates based on cospeciation have used a calibration point at approximately 24 million ybp (the Old World Monkey–ape split, assumed to coincide with the divergence between Herpes simplex and Macacine herpesvirus 1 [Zalmout et al. 2010]). This calibration is over 200 times older than the date of interest, which can cause problems, for example, with saturation of informative sites (Linder et al. 2005; Duchêne et al. 2014). Furthermore, such analyses must rely on the assumption that rates of viral evolution have remained roughly constant over the entire 24-My period.

Another difficulty with dating viral origins is the time-dependency of evolutionary rate estimates (Ho and Larson 2006; Ho et al. 2011; Sharp and Simmonds 2011; Lythgoe and Fraser 2012). This may be a general phenomenon with several causes (Ho et al. 2011), but one factor might be uniquely important in viral evolution, namely viral latency, that is, the ability of pathogenic viruses to lay dormant in the cell. Indeed, several authors have suggested that long periods of latency, with little or reduced viral replication, might reconcile the observations of rapid rates of evolution over short periods and the slower rates implied by plausible biogeographical scenarios (Kelly 1994; Jenkins et al. 2002; Holmes 2003; Switzer et al. 2005; Ramsden et al. 2008; Sharp and Simmonds 2011; Lythgoe and Fraser 2012). This could be an important factor in VZV evolution, as it undergoes periods of latency in neurons (particularly in the trigeminal and dorsal nerves). Indeed, herpes zoster is the result of viral reactivation following latency (Gilden et al. 2011). But although comparative evidence shows that viruses causing latent infection evolve significantly more slowly than viruses with acute or persistent infections (Hanada et al. 2004), we lack evidence that latency is directly affecting evolutionary rates in any single case.

Here, we take a novel approach to investigating the effects of latency on evolutionary rates, by tracking the within-patient evolution of VZV vaccines (Depledge et al. 2013). The vOka vaccine, which was developed in the 1970s (Takahash et al. 1974), is a live attenuated virus. As such, it is free to evolve in patients following their vaccination (Kanda et al. 2011; Quinlivan et al. 2011; Depledge et al. 2013). Vaccination can also cause side effects in some cases. In particular, approximately 5% of vaccinated healthy children experience an attenuated form of the varicella rash (Sharrar et al. 2000; Galea et al. 2008; Goulleret et al. 2010), whereas a smaller number of patients experience latent infection (zoster) as a direct result of the vaccination (Civen et al. 2009).

Within-patient evolution of the VZV vaccine has attracted attention because of its possible role in causing disease (e.g., following reversion toward the wild-type [Quinlivan et al. 2011; Depledge et al. 2013]), but the viral genomes also allow us to study replicated instances of viral evolution over a known time period. Because some of the vaccine genotypes have established latency and reactivated before sampling, the data also allow us to estimate the effects of latency on evolutionary rates.

## Results

### Analysis of Vaccine Batches and Inference of the Ancestral Sequence

To estimate the amount of evolutionary change that has accrued in each patient, we must first infer the genome sequence of the vaccine that was administered to that patient. To this end, we compared whole-genome sequences of different batches of the vOka vaccine (table 1). Specifically, we compared three modern vaccine batches (obtained between 2009 and 2012 in the United States and United Kingdom), to an early batch from 1986. These vaccine batches therefore approximately span the dates of our patient samples (1982–2013; table 1). In an alignment of over 100 kb, we found no fixed differences between any of the vaccine strains, and this remained true when we excluded polymorphic variants segregating at frequencies below 10%.

**Table 1.** Samples used in this study.

Sample	Type	Sequencer	Date	Days	Passage	Acc. No.
VVAG	Vaccine	MiSeq	2009*	—	—	KF558383
VV10	Vaccine	MiSeq	2010	—	—	KF558384
VV12	Vaccine	MiSeq	2012	—	—	KF558385
B86	Vaccine	HiSeq	1986*	—	—	KF853225
A171B	Varicella	GAiix	1988*	14	High	KF853226
A182B	Varicella	GAiix	1988*	16	High	KF558381
A185B	Varicella	GAiix	1988*	21	High	KF558382
O27	Varicella	GAiix	1999*	16	None	KF558376
VR2	Varicella	MiSeq	2007	17	None	KF558372
VR1	Varicella	MiSeq	2007	14	None	KF558373
R43	Varicella	MiSeq	2000*	23	None	KF853229
VR5	Varicella	MiSeq	2008	16	None	KF853233
R3	Zoster	GAiix	1999*	244	Low	KF853228
R52	Zoster	GAiix	1999*	490	Low	KF853230
T17	Zoster	GAiix	2000*	310	Low	KF558378
T25	Zoster	GAiix	2000*	547	Low	KF558379
v76	Zoster	GAiix	1982*	630	Low	KF558380
K11	Zoster	MiSeq	1997*	75	None	KF558391
L53	Zoster	MiSeq	1997*	131	None	KF558389
T61	Zoster	GAiix	2001*	91	None	KF558377
ZR1	Zoster	MiSeq	2006	330	None	KF558371
Q27	Zoster	MiSeq	1998*	541	Low	KF853227
ZR2	Zoster	MiSeq	2006	150	None	KF853231
ZR3	Zoster	MiSeq	2007	577	None	KF853232
ZR4	Zoster	MiSeq	2010	517	None	KF853234
ZR5	Zoster	MiSeq	2013	120	None	KF853235

Note.—Type, either vaccine batch, patient samples from a vaccine varicella rash or vaccine zoster rash; Date, year of vaccine batch production or year of vaccination; Days, time interval between vaccination and rash development; Passage, rates of lab passaging prior to sequencing; Acc. No., GenBank accession numbers of consensus sequence.

This suggests that the process of culturing, by which vaccine batches are derived from a common frozen seed stock, is not introducing substantial evolutionary change, and this suggests that the sequences of the modern vaccine batches will be very close to that administered to the patients. However, all of the vaccine batches did contain genetic variation (supplementary table S1, Supplementary Material online), and so variable vaccine sequences are likely to have been injected into patients (Depledge et al. 2013). Failure to account for such variation could upwardly bias our estimates of evolutionary rates (Nei 1971), although the extent of such an artifact will depend on the profile of the genetic variation within the vaccine.

The bias will be weakest if the genetic variation in the vaccines mostly comprises deleterious mutations. Such variation should contribute little to within-patient evolution, as deleterious mutations segregate at low frequencies, but rarely reach fixation. Previous results suggest that most variation in VZV vaccines is indeed deleterious (Depledge et al. 2013), and so for our major analyses, we used the consensus sequence of the three modern vaccine batches as our assumed ancestral genome.

However, a stronger bias would arise if some of the injected variants were selectively neutral or beneficial once inside the patient (Depledge et al. 2013). Accordingly, to minimize the effects of ancestral polymorphism, we repeated all analyses using only sites that were fixed in all four vaccine batches (including the early batch). Of course, this approach might still miss very low frequency variants, but such variants are expected to have the least effect on rate estimates, essentially because low frequency variants are likely to have arisen only a few viral replications before vaccination (Nei 1971).

A far larger artifact could arise if secondary infections with VZV were mistakenly interpreted as populations that derived from the vaccine. To exclude this possibility, we confirmed that each sample was more closely related to the vOka vaccine strain than to any published genome of wild-type VZV (Zell et al. 2012), and that all carried the diagnostic single nucleotide polymorphisms reported by (Quinlivan et al. 2011). As the vaccine was originally derived from naturally occurring VZV of East Asian origin (Takahash et al. 1974), the patient samples were particularly distantly related to naturally occurring strains from the United States and United Kingdom, where our samples were collected (Kanda et al. 2011; Quinlivan et al. 2011; Zell et al. 2012; Chow et al. 2013; Depledge et al. 2013).

As a final check, we also tested for shared variants between the patient samples, as genuine postvaccination evolution would proceed independently in each patient. We found that the majority of variants observed (118/176 = 67%) were present in no more than a single patient, whereas 95% of the variants were present in fewer than 5 of the 22 strains. This is consistent with our assumption that the evolutionary changes we inferred were indeed accrued after vaccination (see also below).

### Evolutionary Rates in Patients Who Developed Varicella Rashes

For each of our 22 patient samples, we know the length of time between vaccination and sampling of VZV genomes from the resulting rash (table 1). By comparing the genomes from these samples to the (inferred) sequence of the initial vaccine, we estimated the rate of evolution of the attenuated virus within the patient.

**Fig. 1.**
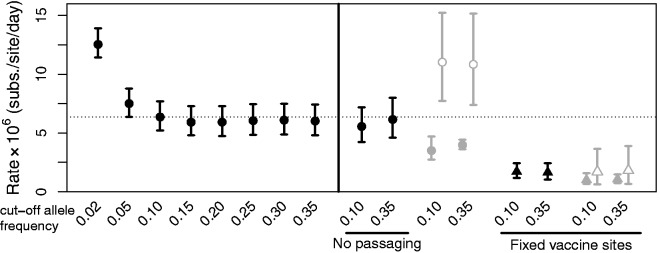
Estimated rates of evolution for the eight VZV strains sampled from patients that had developed varicella rashes after vaccination (table 1). All rates are maximum-likelihood estimates with confidence intervals obtained from the likelihood surface. The left-hand panel shows results varying the allele cut-off frequency (denoted *c* in the supplementary methods, Supplementary Material online), that is, alleles found below the indicated frequency in any single sample were removed from the analysis. The right-hand panel shows how results were affected by removing the three strains that were subjected to passaging in the laboratory (black circles); and by removing any site that was polymorphic in any of the four vaccine batches (triangles). The gray symbols show estimated rates for synonymous changes (empty symbols) and nonsynonymous changes (filled symbols) in protein coding genes.

First, consider the eight samples from patients who developed a varicella rash, and thus were sampled before any viral latency (table 1). Rate estimates can be misled by the inclusion of low frequency polymorphic variants in the patient samples, because these can represent sequencing errors, or mildly deleterious polymorphisms, which violate the assumptions of our rate estimator (e.g., Eyre-Walker 2002; see also supplementary material, Supplementary Material online). Accordingly, we obtained maximum-likelihood estimates with polymorphisms excluded below a range of cut-off frequencies. These estimates are shown in the left-hand panel of figure 1. Rate estimates are clearly substantially higher when polymorphic variants segregating at 5% or below were included in the analysis. This is consistent with evidence that low frequency variants are often sequencing errors (Depledge et al. 2013; supplementary fig. S1, Supplementary Material online). In contrast, estimates were very stable at cut-off frequencies of 10% or above and consistently suggested a rate of approximately 6.36 × 10^−^^6^ substitutions/site/day (CIs: 5.22–7.70 × 10^−^^6^; all rates here and below are reported with the 10% cut-off, unless otherwise specified). Furthermore, rate estimates for all the patient samples were similar (supplementary fig. S2*a*, Supplementary Material online), and there was no evidence of significant between-patient rate variation (Likelihood Ratio Test, Δln *L* = 13.28, df = 7, *P* = 0.07). The constancy of the rate estimates between patients gives us some confidence that the estimates are not being greatly inflated by genetic variation in the vaccine samples, as this artifact would yield a constant amount of apparent evolution, rather than a constant rate, implying a steady increase in evolutionary change with time.

To further determine the robustness of our estimates, we carried out several additional analyses. First, we reestimated the rate after excluding three samples (A171B, A182B, and A185B; table 1) that had been subject to extensive laboratory passaging prior to sequencing. Estimates were little changed (fig. 1), suggesting that neither passaging nor the use of the older GaIIX sequencing technology for some of the samples (table 1) was greatly affecting estimates once low frequency variants were removed (Depledge et al. 2013).

Second, we repeated estimates excluding any site that was found to be polymorphic in any of our four vaccine batches. This is a much more conservative approach to accounting for genetic variation in the vaccine (see above). However, the procedure is also likely to artifactually decrease estimates, by excluding sites under low levels of selective constraint, and so most likely to contribute to evolution. Whatever the cause, including only fixed vaccine sites decreased estimates more than 3-fold to 1.71 × 10^−^^6^ substitutions/site/day (CIs: 1.17–2.43 × 10^−^^6^; fig. 1); and again there was no evidence of variation in rate between patients (Δln *L* = 1.66, df = 7, *P* = 0.97).

Finally, estimates of evolutionary rates over short time periods might be inflated by the inclusion of mutations that are under weak purifying selection, and so unlikely to contribute to evolution over longer time periods (Ho et al. 2011). To test for ineffective purifying selection, we repeated both analyses estimating rates for synonymous and nonsynonymous changes in protein-coding genes (gray symbols in fig. 1). Rates were faster than the genomic average at synonymous sites and slower at nonsynonymous sites, which is consistent with greater levels of purifying selection acting on amino acid changing mutations. And although *d*_n_*/d*_s_ ratios are high (0.59 for all vaccine sites and 0.32 at fixed vaccine sites), they are similar to estimates from the global diversity of VZV (posterior median *d*_n_/*d*_s_ = 0.31, 95% HPD 0.25–0.38; see below), suggesting that purifying selection is effective, despite the short time periods (Ho and Larson 2006).

### Evolutionary Rates in Patients Who Developed Zoster

Results are radically altered when analyses were repeated with the 14 samples from patients who developed herpes zoster (i.e., where vaccine genotypes had undergone latency prior to sampling). Although the qualitative patterns in the data were similar (supplementary fig. S3, Supplementary Material online), the single best-fit rate was between 1 and 2 orders of magnitude slower (∼3.18 × 10^−^^7^ substitutions/site/day with all sites included or ∼1.09 × 10^−^^7^ substitutions/site/day with fixed vaccine sites only). Furthermore, there was very strong evidence of rate variation between patients (Δln *L* = 150.89, df = 13, *P* < 10^−^^6^), and estimates from individual patients varied from 1.10 × 10^−^^7^ to 2.07 × 10^−^^6^ substitutions/site/day (see supplementary fig. S2*b*, Supplementary Material online).

**Fig. 2.**
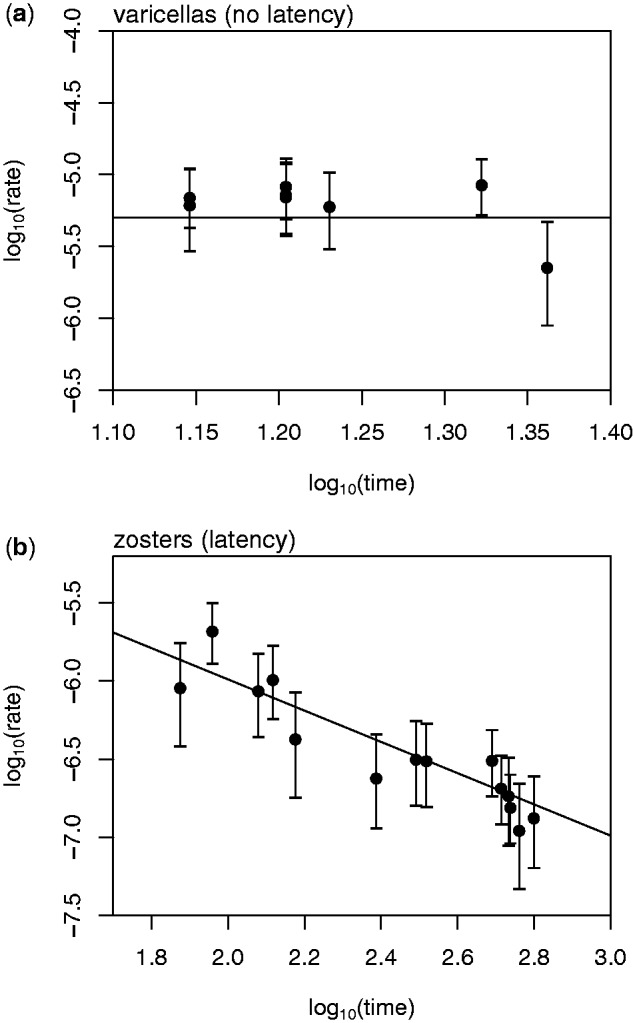
Estimated rates of evolution (substitutions/site/day) for each of the 22 strains of VZV sampled from vaccinated patients. Estimated rates are plotted against the length of time (days) between vaccination and sampling, which followed the appearance of symptoms. Panel (*a*) shows the eight patients who developed varicella rashes, with the horizontal line showing the maximum-likelihood rate for the whole data set. Panel (*b*) shows the 14 patients who developed herpes zoster rashes, with a line with a slope of −1, suggesting that the amount, but not the rate, of evolution is roughly constant in all strains.

Most notably, in contrast to the varicella samples (fig. 2*a*), the estimated rates of evolution for the zoster samples decreased linearly with the length of time between vaccination and the appearance of symptoms (fig. 2*b*). This pattern suggests that the variable rates are due to similar amounts of evolution taking place in each patient, despite the very different times between vaccination and the appearance of herpes zoster. Indeed, model selection with the Bayesian Information Criterion (BIC) suggests that a model with a fixed amount of evolution provides a substantially better fit to the zoster patient data than a model with a fixed rate of evolution or a model with variable rates (BIC values: Single amount 193.8, single rate 318.2, variable rates 219.6).

A natural interpretation for these results is that, following vaccination, the attenuated virus evolves for a fixed amount of time either before and/or after establishing a latent infection. Once latent, replication is arrested so that no new mutations accumulate, regardless of the period of time spent within the host. If this interpretation is correct, then we can estimate this “duration of active viral replication” directly from our data, by assuming that all 22 of our samples evolved at the same rate when they were active (see supplementary material, Supplementary Material online). Taking all 14 zoster samples together, we estimate this duration to be t* = 13.3 days (CIs: 10.9–15.9; individual estimates range between 2.9 and 27.6 days).

### Latency and Dating the Most Recent Common Ancestor of VZV’s Global Diversity

Results above show that changes in viral latency periods can substantially alter evolutionary rates. This implies that rates estimated over very short-term periods might not be reliable if extrapolated back to the origins of VZV. It also questions dates inferred from the complete alphaherpesvirinae phylogeny, which assume that rates remained roughly constant for millions of years before the origin of VZV.

Accordingly, to investigate the global spread of VZV we reanalyzed published whole-genome data, representing the global diversity of the virus (Zell et al. 2012). Because these data were sampled over a 44-year period (1964–2008; see supplementary table S2, Supplementary Material online), temporal signal in the data might be used to date the phylogeny (Rambaut 2000; Firth et al. 2010). If rates of evolution were relatively constant over the sampling period, then we would expect earlier samples to have undergone less molecular evolution. However, analyses reported in the supplementary material, Supplementary Material online, found no evidence of such a pattern (see also Firth et al. 2010), supporting our suggestion that variation in transmission patterns can lead to erratic rate variation in VZV over short time periods.

Because past transmission patterns are unknown to us, it is difficult to date the spread of VZV with much certainty. However, we can ask whether a hypothesized date of the most recent common ancestor is consistent with plausible transmission dynamics.

To do this, we first identified segments of the VZV genome with no evidence of recombination (supplementary fig. S5, Supplementary Material online), and then reconstructed the evolutionary history of these segments using Bayesian phylogenetics, and a relaxed molecular clock. To constrain the evolutionary rate of VZV in these analyses, we used the following equation, which relates short- and long-term rates (i.e., rates with and without latency effects).

(1) $$\mu_{long}=\frac{\mu_{short}t^{*}}{\text{avg. time between infections}},$$

where the numerator is the expected amount of evolutionary change accrued while the virus is replicating, and the denominator is the expected time between infections (including both replication and latency periods). We then made several assumptions to bias our analysis toward an older date estimate: 1) We used the slower of our short-term rate estimates (µ_short_ = 1.71 × 10^−^^6^; fig. 1), obtained by excluding any site that was polymorphic in the vaccine; 2) we used the lower bound of our estimate of the duration of active viral replication (t* = 10.9 days); 3) we assumed an average 50-year gap between transmissions, which is consistent with some observed latency periods (Yawn et al. 2007; Weitzman et al. 2013), but ignores the possibility of varicella-to-varicella transmissions which occur without latency; and 4) we applied our long-term rate solely to third positions in protein-coding genes, thereby allowing other sites in the genome to evolve more slowly.

**Fig. 3.**
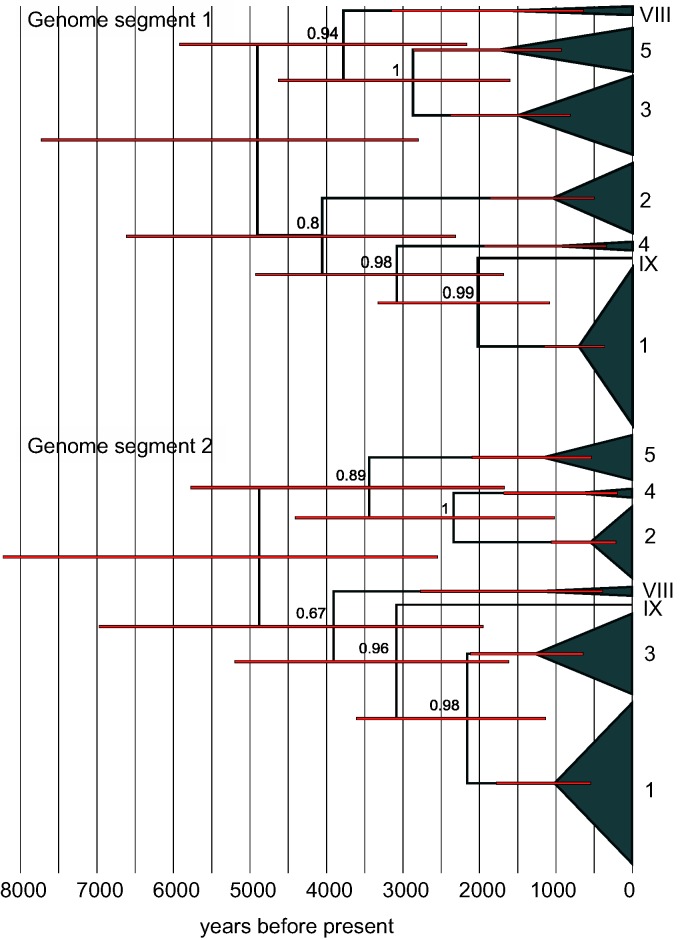
Dated phylogeny of the global diversity of VZV. Shown are Maximum Clade Consensus phylogenies for two large segments of the viral genome that show no evidence of within-segment recombination. Node labels show posterior support values, and red bars represent 95% Credible Intervals on the estimated divergence dates. The labeled clades of VZV (Zell et al. 2012) each appeared with 100% posterior support. Results were obtained after making several conservative assumptions about the evolutionary rate of VZV, nevertheless, the date estimates are substantially younger than is implied by the out-of-Africa hypothesis of VZV origins.

Results of our dating analyses are shown in figure 3. The phylogenies indicate the major clades of VZV, as identified by Zell et al. (2012) (see also supplementary fig. S5, Supplementary Material online), and each phylogeny applies to a different segment of the VZV genome, together comprising approximately 85% of its total length. The phylogenetic topologies of the two genomic segments differ substantially, suggesting that they have recombined since the origins of the virus (McGeoch and Cook 1994; Loparev et al. 2007; Zell et al. 2012). However, the estimated dates of the most recent common ancestors are remarkably similar (segment 1: 4,904 ybp; segment 2: 4,880 ybp). Most importantly, despite our biasing results toward older date estimates, both dates are significantly younger than the youngest possible dates for the out-of-Africa migrations of modern humans (Petraglia et al. 2010).

To confirm this, we repeated our analyses, making no assumptions about long-term rate, but constraining the most recent common ancestor of VZV to 110,000 ybp—the date obtained by Zell et al. (2012), which would be consistent with the out-of-Africa hypothesis. This analysis estimated an average long-term rate of 3.74 × 10^−^^7^ substitutions/site/year. With the foregoing assumptions about short-term rates, a long-term rate this slow would require an average between-transmission time of 1,112 years—which is clearly implausible.

## Discussion

We have studied the evolution of the VZV vaccine within patients. Using the unique biology of VZV, and the availability of a well-characterized live attenuated vaccine, we have shown that viral latency halts the molecular evolution of the virus. This, to our knowledge, is the first direct evidence that latency can reduce evolutionary rates in a single virus species (Holmes 2003; Hanada et al. 2004; Switzer et al. 2005; Sharp and Simmonds 2011; Lythgoe and Fraser 2012).

We have also provided a rate estimate for the vaccine in the absence of latency effects. At approximately 10^−^^3^ substitutions per site per year (∼10^−^^6^ per site per day), our estimates are congruent with some estimates from dsDNA phage (Minot et al. 2013), but are around ten times faster than typical short-term evolutionary rates of dsDNA viruses, as inferred from dated tips dating of between-patient data (supplementary fig. S7, Supplementary Material online). It is possible that viral latency is one cause of this discrepancy, as latency might have occurred in many published viral data sets, but has certainly not occurred for patients who developed varicella rashes following vaccination.

Together, our results have implications for understanding VZV biology. In particular, the strong effect of latency on rates implies that VZV does not return to latency following activation, despite the asymptomatic shedding of VZV in saliva (Nagel, Choe, Cohrs, et al. 2011), and the presence of mRNA transcripts involved in replication in latent cells (Nagel, Choe, Traktinskiy, et al. 2011; Ouwendijk et al. 2012). This failure to return to latency is a probable contrast to herpes simplex virus, a related human alphaherpesvirus (Wilson and Mohr 2012), and is consistent with the hypothesis that replication in the skin or blood is needed for VZV to travel to the nerves (Nagel, Choe, Cohrs, et al. 2011).

Our results also have implications of understanding VZV evolution. First, the effects of latency imply that variation in patterns of transmission will lead directly to variation in evolutionary rates within and between VZV lineages. Evidence suggests that VZV transmission does indeed vary greatly across the world (Yawn et al. 2007; Nichols et al. 2011; Weitzman et al. 2013), and might differ substantially between modern and hunter-gatherer societies. Second, fundamental differences in transmission biology imply that rates of evolution could vary substantially between the alphaherpesvirinae, even when their mutation rate per replication is very similar. Together, this implies that standard methods of molecular dating—whether based on serially sampled genomes or host-virus cospeciation—may be unreliable when applied to global VZV diversity.

Despite this uncertainty, we have argued that the existing data on latency and short-term rates do not support the out-of-Africa scenario for the global spread of VZV (Muir et al. 2002; Petraglia et al. 2010; Grose 2012; Zell et al. 2012). Even making very conservative assumptions, our date estimates for the most recent common ancestor of VZV were substantially younger than required by the out-of-Africa hypothesis (fig. 3). Of course, this conclusion relies on short-term rates estimated during vaccinations, and not from natural transmission chains involving the wild-type virus. Indeed, the only existing rate estimates from a natural transmission chain (from an outbreak in 2000 in Guinea-Bissau, Western Africa) are substantially slower than our vaccine estimates, and instead, are more typical of estimates from other dsDNA viruses (Depledge et al. 2014; supplementary fig. S7, Supplementary Material online). Nevertheless, even with these slower short-term rates (and assuming that no latency was involved in this transmission chain), implausibly large periods of latency would be required to generate the very slow long-term rate (∼10^−^^7^ substitutions/site/year) required by the out-of-Africa hypothesis (Zell et al. 2012; supplementary figs. S4 and S7, Supplementary Material online).

As such, although VZV might well have been present in ancestral African populations, its existing global distribution is unlikely to be explained by patterns of migration from those populations. Instead, current evidence points to a more recent global spread. This possibility is consistent with the topologies shown in figure 3 (where clade 5, which is primarily of African origin [Schmidt-Chanasit and Sauerbrei 2011], is not basal in either tree), and with evidence that aerosol transmission has led to the spread of the virus through single countries over very short periods of time (Hawrami et al. 1997; Quinlivan et al. 2002; Sauerbrei et al. 2011).

## Materials and Methods

### Patient Samples and Genome Sequencing

The samples for this study are listed in table 1, and were obtained from healthy children in the community who were sampled as part of the postsurveillance vaccine studies carried out in the United States (see Sharrar et al. 2000; Galea et al. 2008) and Europe (see Goulleret et al. 2010). Ten of the samples (B86, A171B, R43, VR5, R3, R52, Q27, ZR2, ZR3, ZR4, and ZR5) are new to this study, whereas the remaining 16 were previously described (Depledge et al. 2013). Full details of sequencing library preparation, sequencing, and variant calling are given in the supplementary material, Supplementary Material online.

### Counting Fixed and Polymorphic Differences

To infer the ancestral vaccine sequence, we used two approaches. First, we generated a consensus sequence of the three modern vaccine strains (VVAG, VV10, and VV12; table 1), after excluding all low frequency variants (cut-off frequency 35%) present in each of the samples. Four sites which contained a high frequency variant in all three vaccine batches were excluded from the analysis. Second, we generated a vaccine sequence that excluded any site that was polymorphic at frequency 2% or above in any of the four vaccine batches (table 1); this led to the exclusion of 221 sites. For the patient samples, we used a variety of cut-off allele frequencies (see below). After excluding low frequency variants, sites were scored as polymorphic within the patient if they carried multiple bases, and as fixed differences within the patient if the base(s) differed from that in the ancestral vaccine sequence. All counts of polymorphic and fixed differences are contained in supplementary table S1, Supplementary Material online.

### Maximum-Likelihood Rate Estimation

Rates of evolution are most easily estimated from a single pair of alleles, but each of our patient samples represents a genetically variable population of virions. Methods to estimate evolutionary rates in such cases can be derived from standard population genetics theory, using the joint expectations of the number of polymorphic differences in a sample of alleles, and the number of fixed differences that separate this sample from a known outgroup (Nei 1971; Welch 2006; Depledge et al. 2011). However, existing methods of this kind assume that a known number of alleles have been sequenced, which is not the case with viral samples from patients. As such, to analyze our data, we have developed a new maximum-likelihood estimator of evolutionary rates, which assumes that the number of alleles sampled is large, but unknown, and that variants were excluded below a certain threshold frequency. This method has been implemented in freely available open-source software (see supplementary material, Supplementary Material online, for full details).

### Molecular Dating of VZV Global Diversity

To investigate the origin of VZV, we reanalyzed publicly available complete genome sequences (see supplementary table S2, Supplementary Material online, and references therein). For results reported, we retained all 49 published genomes, but results were qualitatively unaltered when analyses were repeated excluding samples derived from the vOka vaccine stock (Acc. Nos. AB097932 and DQ008354–5), and highly passaged isolates (Acc. Nos. DQ479962–3) (Tyler et al. 2007; Zell et al. 2012). The complete set of genomes yielded an alignment of 126,632 bp with 1,049 segregating sites. Given strong evidence of recombination in VZV (McGeoch and Cook 1994; Loparev et al. 2007; Schmidt-Chanasit and Sauerbrei 2011; Zell et al. 2012), we used phylogenetic tests to identify segments of the VZV genome that appear to have evolved over a single genealogy (Kosakovsky Pond et al. 2006). To date the origin of each genomic segment, we used the Bayesian phylogenetics package BEAST v. 1.7 (Drummond et al. 2012). Full details of the priors and models used in these phylogenetic analyses are given in the supplementary material, Supplementary Material online.

## Supplementary Material

Supplementary material, methods, figures S1–S7, and tables S1 and S2 are available at *Molecular Biology and Evolution* online (http://www.mbe.oxfordjournals.org/).

Supplementary Data
