# Supplementary material for: Rates of Vaccine Evolution Show Strong Effects of Latency: Implications for Varicella Zoster Virus Epidemiology
Source: Mol Biol Evol. 2015 Jan 6;32(4):1020–8. doi: 10.1093/molbev/msu406 (PMC4379407; doi:10.1093/molbev/msu406)
Supplement: Supplementary Data [file supp_32_4_1020__index.html]

Rates of Vaccine Evolution Show Strong Effects of Latency: Implications for Varicella Zoster Virus Epidemiology — Supplementary Data 

# Rates of Vaccine Evolution Show Strong Effects of Latency: Implications for Varicella Zoster Virus Epidemiology

## Supplementary Data

files

**Files in this Data Supplement:**

- Supplementary Data - xlsx file
- Supplementary Data - docx file
- Supplementary Data - xls file
