# Supplementary material for: Rates of Vaccine Evolution Show Strong Effects of Latency: Implications for Varicella Zoster Virus Epidemiology
Source: Mol Biol Evol. 2015 Jan 6;32(4):1020–8. doi: 10.1093/molbev/msu406 (PMC4379407; doi:10.1093/molbev/msu406)
Supplement: Supplementary Data [file supp_msu406_MBE_SI_revision2_final.docx]

**Supporting Information**

**Sequencing library preparation, genome assembly and variant calling**

Our study includes 26 samples of VZV, comprising four vaccine batches and 22 samples from patients who experienced side-effects following vaccination (see Table 1 for details). From each of the 26 samples of VZV vaccine (Table 1), total DNA was extracted using the QiaAMP DNA mini kit (QIAGEN) according to manufacturer’s instructions. DNA quantification was performed using a NanoDrop spectrophotometer and those with 260/280 ratios outside the range 1.7 – 2.1 and 260/230 ratios out the range 1.8 – 2.2 were further purified using the Zymoclean Genomic DNA Clean & Concentrator™ (Zymo Research Corp.). Whole-genome amplification using GenomiPhi V2 (GE Healthcare) was performed using 10ng of starting material where < 50ng total DNA was available (Table 1). Libraries were constructed as per the standard SureSelect XT v1.4 (Agilent) protocol. Enrichment for VZV sequences was performed as described previously ([Depledge *et al.* 2011](#_ENREF_7)). Samples were sequenced across several Illumina platforms (GAIIx, HiSeq and MiSeq) according to availability (Table 1). Because whole-genome amplification has the potential to introduce errors, Figure S1 compares results from a single vaccine sample, with and without amplification. Results confirm that errors are introduced, but that these appear at very low frequencies (explaining why all analyses were repeated with low-frequency variants removed). The robust and unbiased nature of the methodology is further demonstrated in ([Depledge *et al.* 2013](#_ENREF_6)).

Following sequencing, each sample was parsed through QUASR ([Watson *et al.* 2013](#_ENREF_33)) for duplicate removal and read trimming (-q 30, -l 50) and subsequently aligned against the wild-type VZV substrain pOka (Genbank Acc. No. AB097933; ([Gomi *et al.* 2002](#_ENREF_13))) using BWA ([Li, Durbin 2009](#_ENREF_18)). Bases were called so as to recognize that polymorphisms might be present within each patient or vaccine sample. Specifically, alignments were processed using SAMTools ([Li *et al.* 2009](#_ENREF_19)) to generate pileup files for each sample. Consensus sequences for each sample were called with the QUASR module ‘pileupConsensus’ at a variety of cut-off frequencies. These included 2% (equivalent to a single variant base at 50x coverage), and 5%-35% with 5% increments. When multiple bases remained after excluding low frequency variants, suggesting a genetic polymorphism in the sample, these were coded as ambiguities, using the standard IUPAC codes. Variant profiling for each dataset was performed using VarScan v2.2.11 ([Koboldt *et al.* 2012](#_ENREF_15)) with the following parameters: basecall quality ≥ 20, read depth ≥ 50, independent reads supporting minor allele ≥ 2 per strand. In addition, variant calls showing directional strand bias ≥ 0.85 were excluded from further analyses. Iterative repeat regions (R1, R2, R3, R4 and R5) and the terminal repeat region were trimmed prior to analysis, as these cannot be accurately determined using NGS read data. For each sample, the consensus sequence called at 50% (i.e., without ambiguities) has been deposited in GenBank (Acc. nos. in Table 1). After excluding any site where a base was not called in any sample, a final alignment of 101,068bp was made by eye using Se-Al v. 2.0 (Rambaut 1996: Manual sequence alignment software. Available at http://tree.bio.ed.ac.uk/software/seal/). To estimate evolutionary rates in protein-coding genes, analyses were repeated using only the 62 ORFs present in our alignment, according to the annotation of the wild-type pOKA ([Gomi *et al.* 2002](#_ENREF_13)). Twelve pairs of genes contained sections of overlapping reading frame, and these sections were removed, leaving a final alignment of 30,270 codons. All variants were scored as non-synonymous if they changed the amino acid, and as synonymous otherwise. In rare cases where more than one site was variable in a single codon from a single patient sample, we reconstructed the segregating codons parsimoniously, to minimize the number of non-synonymous variants. To estimate substitution rates per site, we calculated the number of non-synonymous and synonymous sites in the vaccine sequence, using the method of Nei and Gojobori ([Nei, Gojobori 1986](#_ENREF_22)).

**Maximum Likelihood rate estimation**

The likelihood of observing our data, given a particular rate of nucleotide substitution, can be obtained by adapting standard population genetics theory. Our data comprise counts of differences from the vaccine strain observed in each patient sample. After excluding polymorphic variants found below a frequency of $0\leq c\leq1$, some of the differences will be fixed and some will be polymorphic. If we begin by assuming that we have sampled a known number, $n$, of alleles from each patient, then the expected number of neutral polymorphisms in our sample is

$E\left[p\right]=S\theta\sum_{i>cn}^{i<(1-c)n} 1/i$ (1)

(e.g., ([Watterson 1975](#_ENREF_34); [Wakeley 2009](#_ENREF_32))), where $S$ is the number of sites, and $\theta=4N_{e}\mu$, where $N_{e}$ is the effective population size, and $\mu$ is the neutral mutation rate per generation. For the fixed differences, if the viral population has been evolving in the patient for $t$ generations, then an expected $S\mu t$ differences will have accrued along the lineage leading to any single allele. However, only mutations occurring before the most recent common ancestor of all of the sampled alleles will be counted as fixed; as such, the expected divergence time must be decreased by an expected $\theta(n-1)/n$ generations (e.g., ([Wakeley 2009](#_ENREF_32))). Furthermore, removing low frequency ancestral polymorphisms might lead us to inflate the number of fixed differences that we count. Combining these three effects, the expected number of fixed differences is found to be

$E\left[d\right]=S\mu t-S\theta\frac{n-1}{n}+S\theta\sum_{i>(1-c)n}^{i=n-1} 1/i$ (2)

For our VZV data, the actual number of alleles in each patient sample is unknown, but, having removed low coverage sites, we can assume that the number of alleles is large. Accordingly, we can take the limit $n\to\infty$ in eqs. (1) and (2), to obtain

$\lambda_{p}\equiv\lim_{n\to\infty} E\left[p\right]=S\theta\ln\left(\frac{1-c}{c}\right)$ (3)

$\lambda_{d}\equiv\lim_{n\to\infty} E\left[d\right]=S\theta t-S\theta\left(1+\ln(1-c)\right)$ (4)

The complete likelihood surface is then obtained by assuming that sites in the genome evolve independently of one another. In this case, the number of differences is Poisson distributed ([Sawyer, Hartl 1992](#_ENREF_24); [Bierne, Eyre-Walker 2004](#_ENREF_2); [Welch 2006](#_ENREF_35)), and we have

$$L=\prod_{i}^{patients} P\left(p_{i},\lambda_{p,i}\right)P\left(d_{i},\lambda_{d,i}\right)$$

where $P\left(k,\lambda\right)=\frac{e^{-\lambda}\lambda^{k}}{k!}$ (5)

Because we know $t$ for each patient, along with $S$ and $c$, we can use eqs. (3)-(5) to obtain maximum likelihood estimates of $\theta$ and $\mu$. Since $t$ will not generally be specified in units of generations, the rate $\mu$ is estimated in the same units as $t$, and, in effect, $\theta$ is scaled by the number of generations per unit time.

This method was used to obtain all of the rate estimates reported, and confidence intervals on the estimates were taken to be the parameter values that reduced the log likelihood by 2 units (e.g., ([Edwards 1992](#_ENREF_11); [Bierne, Eyre-Walker 2004](#_ENREF_2))). To estimate a rate that applied to multiple strains (as in Fig. 1 and Fig. S2), we assumed that the nuisance parameter $\theta$ was common to all strains. However, results were qualitatively unaltered when a distinct $\theta$ was assigned to each strain. To produce Fig. 2 and Fig. S3, each strain was assigned its own $\theta$ and $\mu$ parameters.

To model latency for the zoster strains, we alter the model above by assuming that evolution takes place for a fixed period $\lambda_{d}^{_{(lat)}}=S\theta t^{*}-S\theta\ln\left(1-c\right)$. In this case, the expected number of fixed differences, eq. (4), is replaced by

$\lambda_{d}\equiv\lim_{n\to\infty} E\left[d\right]=S\theta t-S\theta\ln\left(1+\ln(1-c)\right)$ (6)

To compare different parameterizations (some of which are non-nested), we used the Bayesian Information Criterion ([Schwarz 1978](#_ENREF_25)).

$BIC\equiv-2\ln L+K\ln(k)$ (7)

where $K$ is the number of model parameters (i.e., the number of $\theta$, $\mu$ or $\mu t^{*}$ values used to fit the data), and $k$ is the number of data points (taken to be twice the number of patient samples). Models are preferred if they have lower values of the BIC. The likelihood equations used to generate all of our results involve some important simplifying assumptions. For example, linkage between sites will inflate the variance above eq. (5); however, this assumption will not affect the maximum likelihood estimates when each patient is assigned their own value of $\theta$ and $\mu$ (as in Fig. 2), because it does not affect the expected values, eqs. (3)-(4) and (6). Also, the equations assume that no balancing selection or weak purifying selection is acting on the polymorphic variants. Unless balancing selection is common, the greatest problems are likely to arise from weakly deleterious variants, which segregate at low frequencies. As such, the robustness of our estimates to varying the cut-off frequencies (Fig. 1), gives us some confidence that weakly deleterious polymorphisms are not unduly affecting our results. The extension of the model to synonymous sites (Figs. 1 and S2) is relatively simple, and requires that we include two new terms in the likelihood, accounting for non-synonymous and synonymous divergence and polymorphism (see ([Welch 2006](#_ENREF_35)) for full details). The number of synonymous and non-synonymous sites were calculated for our data using the method of Nei and Gojobori ([Nei, Gojobori 1986](#_ENREF_22)). All likelihood estimation was undertaken by implementing the new models in the MKtest software of ([Welch 2006](#_ENREF_35)), which is written in C and estimates the parameters using a method of simulated annealing. The software is freely available at sitka.gen.cam.ac.uk/research/welch/GroupPage/Software.html.

**Analysis of global VZV data set**

The methods described in the following section apply to our reanalyzes of published whole-genome data from VZV (see Table S2 for details). These data comprise the 49 complete VZV genomes available on GenBank (www.ncbi.nlm.nih.gov/genbank/), which were aligned by eye using Se-Al v. 2.0. To identify regions of these genomes which evolved over a single genealogy, we first analyzed an alignment of the segregating sites in the Bayesian recombination detection program GARD ([Kosakovsky Pond *et al.* 2006](#_ENREF_16)), as implemented on the *Datamonkey* server ([Delport *et al.* 2010](#_ENREF_4)). This software searches for breakpoints in the alignment that separate regions with significant topological incongruity, and so different evolutionary histories. It does not aim to identify weakly supported potential breakpoints, or to estimate the overall rate of recombination. The GARD analysis gave clear evidence of two well-supported breakpoints (Fig. S5), dividing the genome into three segments. Using the annotation of isolate 3/2005 of ([Zell *et al.* 2012](#_ENREF_37)); Genbank Acc. No. JN704700), the first breakpoint falls within ORF 29, between polymorphic sites 46183 and 46477, and the second breakpoint falls between ORFs 62 and 63, between the polymorphic sites 104799 and 104854. This analysis, as shown in Fig. S5, used the HKY model of nucleotide substitution, but results were unchanged when a HKY+Γ model was specified, allowing for rate variation among sites. All subsequent analyses were carried out on individual genomic segments. To test for temporal signal in the data, we used the prediction that, with relatively constant rates of evolution, we expect a positive correlation between sampling dates, and root-to-tip branch lengths (with more recently sampled sequences having undergone more molecular evolution). We tested for such a correlation in the global VZV data set, using the software Path-O-Gen, v. 1.4 (Rambaut, A. 2013: Available at http://tree.bio.ed.ac.uk/software/pathogen/), applying the test separately to each of the genomic segments identified by GARD (see Methods). Even after choosing the root to maximize the correlation, no indication of temporal signal was found in any of the three genomic segments. Indeed the slopes were weakly negative in two cases (segment 1: *slope*= -0.0009 , *r*^2^ = 0.192 ; segment 2: *slope*=-0.0005 , *r*^2^ = 0.157; segment 3: *slope*=2.41×10^-4^, *r*^2^ = 5.30×10^-3^). The results of our Path-O-Gen analysis are consistent with the randomization test of ([Firth *et al.* 2010](#_ENREF_12)), and both suggest that the method of dated tips cannot be used to date the phylogeny of VZV. This is consistent with our finding that variation in latency period might alter evolutionary rates over short periods of time. The molecular dating analyses used the Bayesian phylogenetics package BEAST v. 1.7 ([Drummond *et al.* 2012](#_ENREF_9)). Separate analyses were carried out each genomic segment (as identified by GARD) and all tips were treated as contemporaneous (consistent with the lack of temporal signal in the data). Because we are particularly interested in the evolutionary rate of relatively unconstrained sites (which are less likely to show time-dependent rates due to ineffective purifying selection), and in the relative rates of non-synonymous and synonymous changes in protein coding genes, we divided each of our alignments into three partitions: (*i*) third codon positions in non-overlapping reading frames, (*ii*) first and second coding positions in non-overlapping reading frames, (*iii*) and all other sites. Protein-coding genes, and regions of overlapping reading frame, were identified using the annotation of isolate 3/2005 of Zell et al. ([Zell *et al.* 2012](#_ENREF_37)) (Acc. No. JN704700), and the resulting alignment of protein-coding genes from each strain was checked by eye. Each of the three partitions was assigned its own HKY+Γ model of nucleotide substitution and its own relative rate of evolution. To allow for heterogeneity in rates across branches, we used the uncorrelated lognormal model ([Drummond *et al.* 2006](#_ENREF_8)), a fixed-size coalescent prior was placed on node ages, and all other priors were assigned to their defaults according to BEAUti v. 1.7 ([Drummond *et al.* 2012](#_ENREF_9)). For the analyses with constrained rates (Figure 3), we applied a lognormal prior to the rates on partition (*i*), the 3rd codon positions. Parameters of the lognormal distribution were chosen such that the mean log rate was -14.80017 and the standard deviation in log rate was 0.2. These parameters were chosen such that median and 95% quantiles of the prior distribution of rates matched the values reported in the main text, accounting for uncertainty in the estimate of the short-term rate (Fig. 1). For the analyses with a constrained age of the most recent common ancestor, we removed the prior on rates, and included a normal prior on the date of the root node. This normal prior had a mean of 110,000 ybp ([Zell *et al.* 2012](#_ENREF_37)), and with a very small variance,$\sigma/\mu=10^{-4}$. For each analysis, two MCMCs were run for 10^8^ iterations, and chains were checked for convergence and length of burnin using Tracer v 1.4 (Rambaut and Drummond 2007: Available from http://beast.bio.ed.ac.uk/Tracer). Convergence was always successful for the two larger genomic segments, but never successful for the much shorter third segment (Fig. 5A), which comprised ~15% of the genome. Furthermore, the major clades of VZV (Fig. 3 and Fig. S6) did not appear as monophyletic clades in the analyses of the third segment, while they were present with 100% posterior support in the analyses of the two larger segments (Fig. 3). These results are all suggestive of undetected fine-scale recombination within the third genome segment, and so the main test reports results solely for the two larger genomic segments.

**Supporting Information**

*Figure S1*

The effects of genome amplification on sequencing error rates are shown. The vaccine batch VV12 (Table 1), was split into two, with one portion sequenced after standard library preparation and enrichment, and the other undergoing whole-genome amplification prior to library preparation and enrichment. Each panel shows the relative frequencies of variant alleles in the two datasets. The left-hand panels show the complete data set, while the right-hand panels show the same data after removing alleles observed below a cut-off frequency of 2.5%. Lower panels present the same data as the upper panels, with log-scaled axes to better profile differences at low frequency. Together, the plots show that discrepancies do exist, but that very few remain after very low frequency variants are removed.

*Figure S2*

Estimated rates of evolution for each of the 22 VZV strains. Estimates are shown with allele frequency cut-offs of 10% (darker bars) and 35% (lighter bars). Panel (a) shows the 8 patients who developed attenuated varicella rashes, and panel (b) shows the 14 patients who developed zosters. Noting the different y-axes, it is clear that estimated rates for the zoster patients (b) are consistently lower, and show much more variation between strains (see text).

*Figure S3*

Estimated rates of evolution for the 14 VZV strains sampled from patients that had developed zosters after vaccination (Table 1). All other details are as for Figure 1.

*Figure S4*

Comparison of evolutionary rate estimates for the VZV. Shown are rates of within-patient vaccine evolution estimated from the 8 patients who developed varicella rashes, and from each of the 14 patients who developed zosters. These rates are compared for all sites in the genome (filled circles) and sites that were fixed in all four vaccine strains (open circles). The following points show rates estimated from the global diversity of VZV, as estimated from published genome sequences. Shown are rates at all sites (black), at first and second positions in protein coding genes (grey circles) and at third codon positions (white). These rates were estimated by assuming a root age of 110,000 ybp([Zell *et al.* 2012](#_ENREF_37)), consistent with the out-of-Africa hypothesis. Also shown, are rate estimates from previous studies, including Firth *et al.*([Firth *et al.* 2010](#_ENREF_12)), who used the method of dated tips; and Zell *et al.* ([Zell *et al.* 2012](#_ENREF_37)) and Muir *et al.* ([Muir, Nichols, Breuer 2002](#_ENREF_21)); see also ([McGeoch, Cook 1994](#_ENREF_20))), who assumed cospeciation between mammals and alphaherpesviridae.

*Figure S5*

Support for two recombination breakpoints in the VZV virus genome, as estimated with the Bayesian recombination detection software GARD ([Kosakovsky Pond *et al.* 2006](#_ENREF_16)). Each of the three shaded regions, separated by the breakpoints, supported a significantly different phylogenetic topology for the global diversity of VZV. The *x*-axis corresponds to the length of the genome, and is measured in segregating sites. However, in terms of the annotated genome Acc. No. JN704700, the breakpoints fall between sites 46183 and 46477 (first breakpoint), and 104799 and 104854 (second breakpoint).

*Figure S6*

Estimated rates of evolution for the different strains of VZV (supplementary Table S2). Rate estimates (median and 95% credible interval) are shown for the terminal branches of the Bayesian Maximum Clade Consensus phylogenies depicted in Figure 3, with estimates from genome segment 1 shown as circles, and genome segment 2 shown as triangles. Shaded areas indicate the major clades of VZV indicated in Figure 3. The vaccine strains (found in clade 2) are highlighted in red. Results confirm that the rates of evolution along lineages leading to the vaccine strains do not differ significantly from the rates in the wild-type lineages.

*Figure S7*

Comparison of our vaccine rate estimates to several published estimates from wild DNA viruses, obtained from the method of dated tips. Such estimates are reliable only when the data contain temporal signal, and we have excluded estimates where failed tests were reported in the source publications. All estimates are shown as substitutions per site per year, and are posterior means with 95% credible intervals. Estimates from a wild VZV infection from Guinea-Bissau correspond to the complete data set of ([Depledge *et al.* 2014](#_ENREF_5)), and the “5B genogroup”. Other double-stranded DNA viruses are Herpesviridae: HSV (Herpes simplex virus ([Firth *et al.* 2010](#_ENREF_12))) CFPHV (Chelonid fibropapilloma-associated herpesvirus ([Patrício *et al.* 2012](#_ENREF_23))); Adenoviridae: HAdV (human adenovirus ([Firth *et al.* 2010](#_ENREF_12))); Papillomaviridae: HPV (Human papillovirus ([Firth *et al.* 2010](#_ENREF_12))); Polyomaviridae: BKV (B. K. virus ([Chen *et al.* 2004](#_ENREF_3); [Firth *et al.* 2010](#_ENREF_12))); JCV (John Cunningham virus ([Shackelton *et al.* 2006](#_ENREF_28))); Poxviridae: VARV (variola or smallpox virus([Firth *et al.* 2010](#_ENREF_12))). Single-stranded DNA viruses are Anelloviridae: SENV (SEN-virus ([Umemura *et al.* 2002](#_ENREF_31))); Geminiviridae: EACMV (East Africa cassava mosaic virus) MSV (Maize streak virus) SSRV (Sugarcane streak reunion virus([Lefeuvre *et al.* 2011](#_ENREF_17))) TYLCV (Tomato yellow leaf curl virus ([Duffy, Holmes 2008](#_ENREF_10); [Lefeuvre et al. 2011](#_ENREF_17))); Parvoviridae: B19 (Primate erythroparvovirus 1 ([Shackelton, Holmes 2006](#_ENREF_26))); CPV (Canine parovirus); FPV (Feline panleukopenia virus ([Shackelton et al. 2005](#_ENREF_27); [Hoelzer et al. 2008](#_ENREF_14))); PPV (Porcine parovirus([Streck *et al.* 2011](#_ENREF_29))). Double-stranded DNA viruses with an RNA intermediate, are represented by HBV (Hepatitis B virus ([Zhou, Holmes 2007](#_ENREF_38); [Alvarado-Mora et al. 2010](#_ENREF_1); [Torres et al. 2011](#_ENREF_30); [Xu et al. 2013](#_ENREF_36))).

*Table S1*

Number of polymorphic and fixed differences accrued in the rash samples compared to the vaccine consensus sequence and fixed vaccine sites, at different polymorphic cut-off frequencies.

*Table S2*

Details of published whole genomes used to investigate the global spread of VZV.

**Supplementary References**

Alvarado-Mora, MV, CM Romano, MS Gomes-Gouvea, MF Gutierrez, FJ Carrilho, JR Pinho. 2010. Molecular epidemiology and genetic diversity of hepatitis B virus genotype E in an isolated Afro-Colombian community. Journal of General Virology 91:501-508.

Bierne, N, A Eyre-Walker. 2004. The genomic rate of adaptive amino acid substitution in Drosophila. Molecular Biology and Evolution 21:1350-1360.

Chen, Y, PM Sharp, M Fowkes, O Kocher, JT Joseph, IJ Koralnik. 2004. Analysis of 15 novel full-length BK virus sequences from three individuals: evidence of a high intra-strain genetic diversity. Journal of General Virology 85:2651-2663.

Delport, W, AFY Poon, SDW Frost, SLK Pond. 2010. Datamonkey 2010: a suite of phylogenetic analysis tools for evolutionary biology. Bioinformatics 26:2455-2457.

Depledge, DP, ER Gray, S Kundu, S Cooray, A Poulson, P Aaby, J Lockwood, J Breuer. 2014. Evolution of viral genomes during a varicella outbreak in Guinea Bissau. submitted.

Depledge, DP, S Kundu, N Jensen, et al. 2013. Deep sequencing of viral genomes provides insight into the pathogenesis of varicella zoster virus and its vaccine in humans. Molecular Biology and Evolution *In press*.

Depledge, DP, AL Palser, SJ Watson, IY-C Lai, ER Gray, P Grant, RK Kanda, E Leproust, P Kellam, J Breuer. 2011. Specific Capture and Whole-Genome Sequencing of Viruses from Clinical Samples. Plos One 6:e27805.

Drummond, AJ, SYW Ho, MJ Phillips, A Rambaut. 2006. Relaxed phylogenetics and dating with confidence. Plos Biology 4:699-710.

Drummond, AJ, MA Suchard, D Xie, A Rambaut. 2012. Bayesian Phylogenetics with BEAUti and the BEAST 1.7. Molecular Biology and Evolution 29:1969-1973.

Duffy, S, EC Holmes. 2008. Phylogenetic evidence for rapid rates of molecular evolution in the single-stranded DNA begomovirus tomato yellow leaf curl virus. Journal of Virology 82:957-965.

Edwards, AWF. 1992. Likelihood : expanded edition. Baltimore [u.a.]: Johns Hopkins Univ. Press.

Firth, C, A Kitchen, B Shapiro, MA Suchard, EC Holmes, A Rambaut. 2010. Using Time-Structured Data to Estimate Evolutionary Rates of Double-Stranded DNA Viruses. Molecular Biology and Evolution 27:2038-2051.

Gomi, Y, H Sunamachi, Y Mori, K Nagaike, M Takahashi, K Yamanishi. 2002. Comparison of the complete DNA sequences of the Oka varicella vaccine and its parental virus. Journal of Virology 76:11447-11459.

Hoelzer, K, LA Shackelton, CR Parrish, EC Holmes. 2008. Phylogenetic analysis reveals the emegence, evolution and dispersal of carnivore paroviruses. Journal of General Virology 89:2280-2289.

Koboldt, DC, Q Zhang, DE Larson, D Shen, MD McLellan, L Lin, CA Miller, ER Mardis, L Ding, RK Wilson. 2012. VarScan 2: Somatic mutation and copy number alteration discovery in cancer by exome sequencing. Genome Research 22:568-576.

Kosakovsky Pond, SL, D Posada, MB Gravenor, CH Woelk, SDW Frost. 2006. GARD: a genetic algorithm for recombination detection. Bioinformatics 22:3096-3098.

Lefeuvre, P, GW Harkins, JM Lett, RW Briddon, MW Chase, B Moury, DP Martin. 2011. Evolutionary Time-Scale of the Begomoviruses: Evidence from Integrated Sequences in the Nicotiana Genome. PLoS ONE 6:e19193.

Li, H, R Durbin. 2009. Fast and accurate short read alignment with Burrows-Wheeler transform. Bioinformatics 25:1754-1760.

Li, H, B Handsaker, A Wysoker, T Fennell, J Ruan, N Homer, G Marth, G Abecasis, R Durbin. 2009. The Sequence Alignment/Map format and SAMtools. Bioinformatics 25:2078-2079.

McGeoch, DJ, S Cook. 1994. Molecular phylogeny of the alphaherpesvirinae subfamily and a proposed evolutionary timescale. Journal of Molecular Biology 238:9-22.

Muir, WB, R Nichols, J Breuer. 2002. Phylogenetic analysis of varicella-zoster virus: Evidence of intercontinental spread of genotypes and recombination. Journal of Virology 76:1971-1979.

Nei, M, T Gojobori. 1986. Simple methods for estimating the numbers of synonymous and nonsynonymous nucleotide substitutions. Molecular Biology and Evolution 3:418-426.

Patrício, AR, LH Herbst, A Duarte, X Vélez-Zuazo, N Santos Loureiro, N Pereira, L Tavares, GA Toranzos. 2012. Global phylogeography and evolution of chelonid fibropapilloma-associated herpesvirus. Journal of General Virology 93:1035-1045.

Sawyer, SA, DL Hartl. 1992. Population genetics of polymorphism and divergence. Genetics 132:1161-1176.

Schwarz, G. 1978. Estimating the Dimension of a Model. Annals of Statistics 6:461-464.

Shackelton, LA, EC Holmes. 2006. Phylogenetic evidence for the rapid evolution of human B19 erythrovirus. Journal of Virology 80:3666-3669.

Shackelton, LA, CR Parrish, U Truyen, EC Holmes. 2005. High rate of viral evolution associated with the emergence of carnivore parovirus. Proceedings of the National Academy of Sciences USA 102:379-384.

Shackelton, LA, A Rambaut, OG Pybus, EC Holmes. 2006. JC virus evolution and its association with human populations. Journal of Virology 80:9928-9933.

Streck, AF, SL Bonatto, T Homeier, CK Souza, KR Goncalves, D Gava, CW Canal, U Truyen. 2011. High rate of viral evolution in the capsid protein of porcine parvovirus. Journal of General Virology 92:2628-2636.

Torres, C, FG Pineiro y Leone, SC Pezzano, VA Mbayed, RH Campos. 2011. New perspectives on the evolutionary history of hepatitis B virus genotype F. Molecular Phylogenetics and Evolution 59:114-122.

Umemura, T, Y Tanaka, K Kiyosawa, HJ Alter, JW Shih. 2002. Observation of positive selection within hypervariable regions of a newly identified DNA virus (SEN virus). FEBS Letters 510:171-174.

Wakeley, J. 2009. Coalescent theory : an introduction. Greenwood Village, Colo.: Roberts & Co. Publishers.

Watson, SJ, MRA Welkers, DP Depledge, E Coulter, JM Breuer, MD de Jong, P Kellam. 2013. Viral population analysis and minority-variant detection using short read next-generation sequencing. Philosophical Transactions of the Royal Society B-Biological Sciences 368.

Watterson, GA. 1975. On the number of segregating sites in genetical models without recombination. Theoretical Population Biology 7:256-276.

Welch, JJ. 2006. Estimating the genomewide rate of adaptive protein evolution in Drosophila. Genetics 173:821-837.

Xu, G, C Wei, Y Guo, C Zhang, N Zhang, G Wang. 2013. An analysis of the molecular evolution of Hepatitis B viral genotypes A/B/D using a Bayesian evolutionary method. Virology Journal 10:256.

Zell, R, S Taudien, F Pfaff, P Wutzler, M Platzer, A Sauerbrei. 2012. Sequencing of 21 Varicella-Zoster Virus Genomes Reveals Two Novel Genotypes and Evidence of Recombination. Journal of Virology 86:1608-1622.

Zhou, Y, EC Holmes. 2007. Bayesian estimations of the evolutionary rate and age of hepatitis B virus. Journal of Molecular Evolution 65.
